# Endoscopic Removal of Multiple Ingested Cylindrical Batteries

**DOI:** 10.7759/cureus.21789

**Published:** 2022-01-31

**Authors:** Sotaro Ozaka, Kunimitsu Inoue, Takako Tasaki, Hideki Ono, Kazunari Murakami

**Affiliations:** 1 Department of Gastroenterology, Faculty of Medicine, Oita University, Yufu, JPN; 2 Department of Gastroenterology, Almeida Memorial Hospital, Oita, JPN

**Keywords:** endoscopy, foreign body ingestion, clinical case report, digestive endoscopy, cylindrical battery

## Abstract

Button battery ingestion accidents have been reported in multiple previous reports. However, ingestion of cylindrical-type batteries is significant less described in the literature. Cylindrical batteries can reportedly cause corrosive damage to the gastrointestinal mucosa after long-term retention, leading to ulceration and perforation. Here, we present a case of endoscopic removal of eight AA batteries that had been ingested and caused corrosive changes in the gastrointestinal mucosa. A 45-year-old man with mental retardation was brought to our hospital due to the suspicion of cylindrical battery ingestion. A plain abdominal x-ray revealed a total of eight cylindrical batteries. Esophagogastroduodenoscopy was performed approximately 24 hours after ingestion, and four AA batteries were removed using a polypectomy snare. The remaining four batteries were followed up and removed under colonoscopy after confirming that they had reached the rectum. Leaked components of retained cylindrical batteries can cause chemical mucosal damage in the gastrointestinal tract. Therefore, early extraction should be considered in case of cylindrical battery ingestion. On the other hand, when the cylindrical battery has passed the pyloric ring, conservative management with close monitoring is acceptable if there are no clinical symptoms. Additionally, a polypectomy snare is useful in the extraction of ingested cylindrical batteries.

## Introduction

Button battery ingestion accidents have been reported in multiple previous reports. However, ingestion of cylindrical-type batteries has been significantly less often described in the current literature [1]. Therefore, no clear practice guidelines have been established for the management of cylindrical battery ingestion. Although current guidelines recommend waiting 48 hours after ingestion to remove cylindrical batteries, a recent report has suggested that cylindrical batteries should be removed urgently [2], because they can cause corrosive and toxic damage to the mucosa if they remain in the gastrointestinal tract for a long time, due to leakage of their contents [1]. Cylindrical batteries have been reported to cause significant gastric ulceration, especially when they are retained in the stomach [2-4]. On the other hand, cylindrical batteries that pass through the pyloric ring into the small intestine are often followed up with the expectation of spontaneous expulsion [5-7]. With regard to the method of cylindrical battery extraction, previous reports have variously described the use of nets, snares, or baskets.

In this article, we report a case of endoscopic removal of eight AA batteries that were ingested and caused corrosive changes in the gastrointestinal mucosa, four from the stomach under esophagogastroduodenoscopy (EGD), and the remaining four that had passed beyond the pyloric ring were removed from the rectum under colonoscopy after follow-up, using a snare in both procedures.

## Case presentation

**Figure 1:**
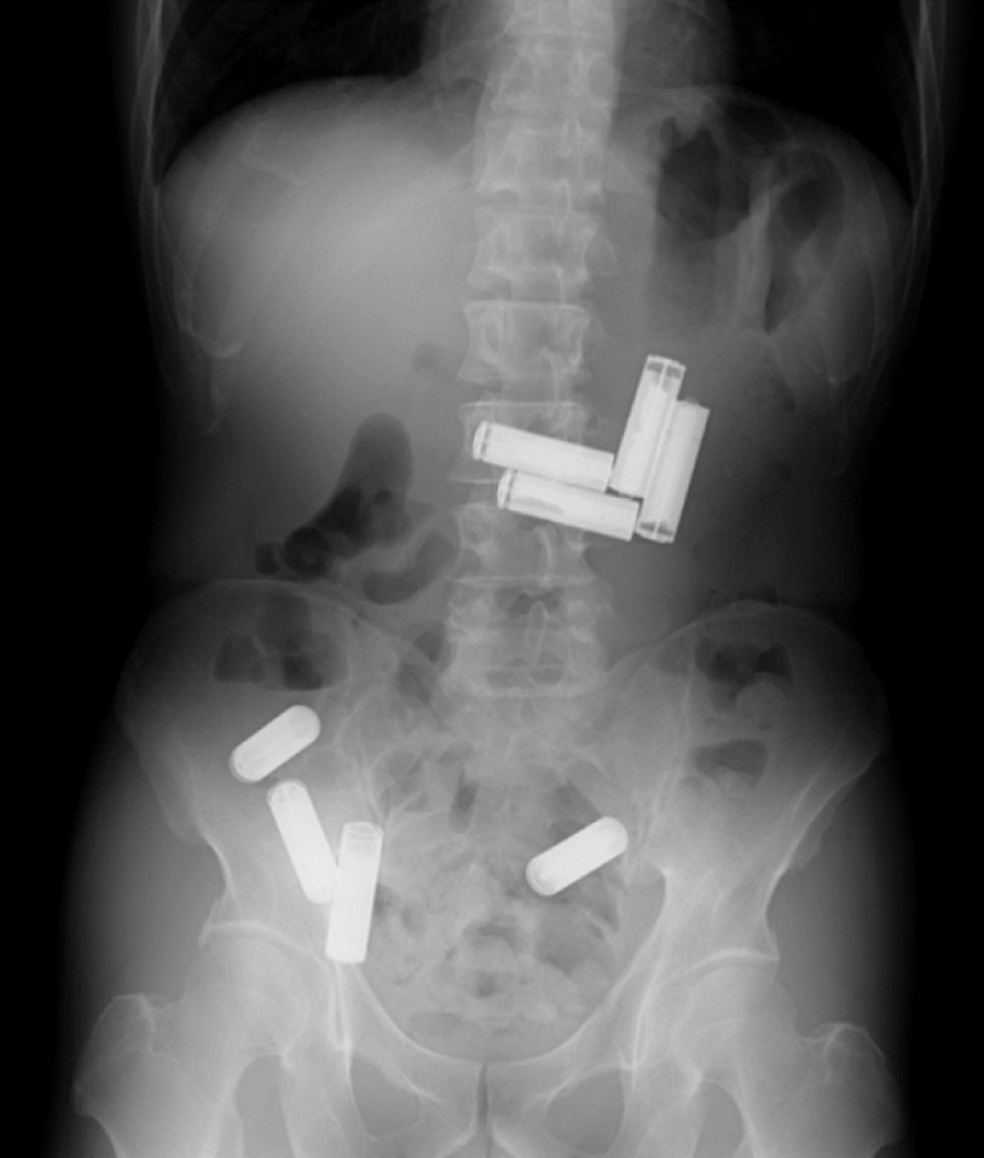
Plain abdominal x-ray showing eight cylindrical batteries in the left upper and lower abdomen.

A 45-year-old man with mental retardation was brought to our emergency department from a psychiatric facility due to the suspicion that he had ingested cylindrical batteries since all the cylindrical batteries in his room were missing. He had undergone colonoscopy at another hospital one week prior to the current presentation for further examination of positive fecal occult blood, at which time two AAA batteries had been removed. He was asymptomatic and his vital signs were normal. On physical examination, his abdomen was soft and non-tender, with no masses, and bowel sounds were normal. Laboratory data were unremarkable. A plain abdominal x-ray showed eight cylindrical structures in the left upper and lower abdomen, consistent with battery ingestion (Figure 1).

**Figure 2:**
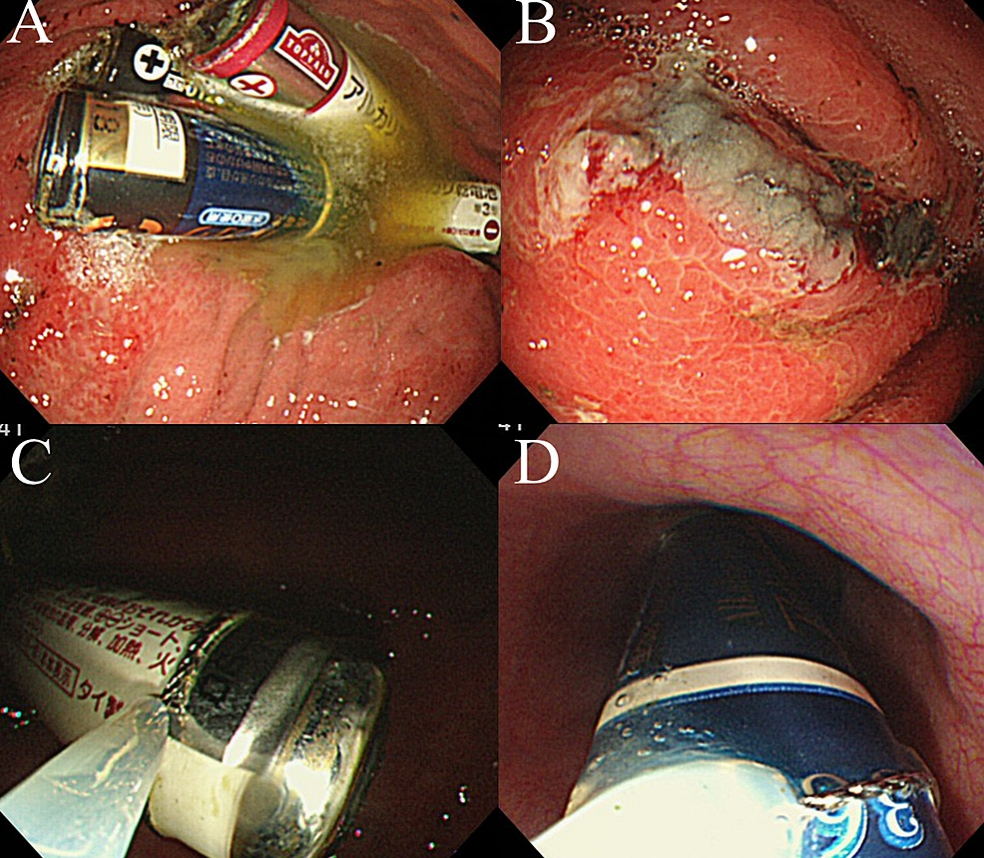
Endoscopic images. (A) Endoscopic images of the four AA batteries in the stomach. (B) Scattered hemorrhagic erosions with black discoloration were observed in the mucosa of the gastric body. (C, D) Endoscopic view showing an AA battery removed using a polypectomy snare.

There was no evidence of perforation or obstruction. EGD was performed approximately 24 hours after the suspected ingestion, which revealed four AA batteries in the stomach. Moreover, there were scattered hemorrhagic erosions with black discoloration of the mucosa from the greater curvature of the middle part of the body of the stomach to the greater curvature of the fornix. The remaining four batteries were not visible on EGD up to the transverse part of the duodenum. The initial approach was to remove the AA batteries using a Roth net, but this was found to be difficult because the battery (14.2 mm diameter, 50 mm in length) was too large to be contained in the net (30 × 50 mm). Next, we used a polypectomy snare, which enabled easy extraction by holding the edge of the battery tightly with the snare and aligning the axis of the battery with the long axis of the esophagus (Figures 2A-2D).

**Figure 3:**
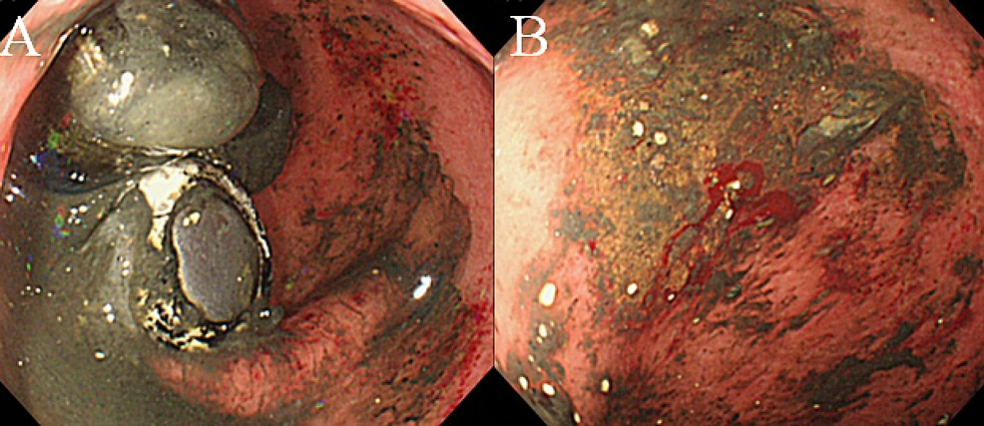
Endoscopic views of the rectum. (A) Colonoscopy showed four AA batteries in the lower rectum, with contents leaking from some of the batteries. (B) The leaked contents of the battery caused hemorrhagic erosion in the rectum.

A total of four AA batteries were removed one at a time in this manner. The time required for the removal of a single AA battery was approximately five minutes. Since the patient was asymptomatic, the remaining four batteries, which had already moved deeper into the duodenum, were followed up with imaging examinations. During follow-up, the patient fasted, and abdominal plain x-ray images were taken every 24 hours to monitor the movement of the batteries. No new clinical symptoms were observed during this period. On hospital day 3, all four batteries were confirmed to have reached the rectum. Since the size of the battery was large and it was expected to take time to pass the anus, we decided to perform foreign body retrieval under colonoscopy. Colonoscopy showed the four AA batteries in the lower rectum, with contents leaking from some of the batteries. All four batteries were removed using a polypectomy snare. After removal of the batteries, the rectum was examined and some easy-bleeding mucosa was identified (Figures 3A, 3B).

The patient resumed eating on hospital day 4 and was subsequently discharged to a residential facility without any abdominal symptoms. Plain abdominal x-ray 10 days after discharge showed no remaining batteries in his gastrointestinal tract.

## Discussion

Our experience with this patient indicated the following clinical issues with such cases. Since cylindrical batteries cause chemical mucosal damage when retained in the stomach, their early removal is necessary. However, if the cylindrical battery moves beyond the pyloric ring into the small intestine, conservative management with close monitoring is also permissible. Additionally, polypectomy snares might be useful tools for the removal of ingested cylindrical batteries.

Although many cases of accidental ingestion of button batteries have been previously reported, there are few reports of accidental ingestion of cylindrical batteries. Litovitz et al. reported that of 8,648 cases of battery ingestion from 1990 to 2008 in the United States, 8,161 (94.4%) involved a button battery and 487 (5.6%) were cylindrical batteries [8]. Most cases of accidental ingestion of a button battery occur in children [9], whereas ingestion of a cylindrical battery is seen more commonly due to deliberate ingestion in a psychiatric population and by prisoners [10]. Our patient also had mental retardation as the underlying disease.

Since cylindrical batteries cause corrosive and toxic damage to the mucosa when they remain in the gastrointestinal tract, their early extraction is necessary. Guidelines issued by the American Society for Gastrointestinal Endoscopy recommend extraction when cylindrical batteries remain in the stomach for over 48 hours after ingestion [11]. However, cases of significant gastric ulceration and gastritis within 12 hours after ingestion [2] and deep gastric ulcers within about 24 hours [3,4] have been reported. Our case also showed corrosive changes in the gastric mucosa about 24 hours after ingestion. Therefore, endoscopic removal should be considered as early as possible if a cylindrical battery is retained in the stomach, due to the increased risk of perforation. In other previous reports, a cylindrical battery that had moved beyond the pyloric ring into the small intestine was surgically removed, although, in recent years, most of the cases were followed up with the expectation of spontaneous elimination [5-7]. Hammami et al. reviewed 15 cases and one case series of cylindrical battery ingestion and reported that no major complications occurred in asymptomatic cases at initial presentation, despite choosing conservative management [10]. Therefore, it is suggested that conservative management with close clinical monitoring might be possible if the patient is asymptomatic and the ingested battery cannot be seen by EGD. However, it is important to note that there have been reports of cases of retained cylindrical batteries in the intestine causing intestinal obstruction or perforation [12,13]. If the battery remains in one place or if abdominal symptoms are observed, surgical intervention should be considered immediately. Since the present case was asymptomatic, the four batteries that could not be confirmed by EGD were followed up with imaging examinations and were removed by colonoscopy without complications.

In our case, a polypectomy snare was useful for the removal of the cylindrical batteries. Since cylindrical batteries are large and hard, and their surface is round and slippery, ingenuity is required for their removal. We summarize the case reports of cylindrical batteries that were removed by endoscopy in Table 1.

**Table 1:** Case reports of cylindrical batteries ingestion successfully removed by endoscopy.

Case	Ref.	Author/Year	Age/Sex	Interval between ingestion and diagnosis	Number and type of batteries	Device	Endoscopic findings
1	[14]	Young/1989	33/M	6 hours	5 AAA	Basket	Undamaged mucosa
2	[15]	Lim/006	60/M	NA	2 Duracell 3-volt	Net	Multiple deep ulcer
3	[16]	Moriyama/2006	42/M	24 hours	3 AAA	Net	NA
4	[17]	Imai/2007	13/M	78 hours	14 AA	Snare, magnet catheter	Multiple ulcer
5	[3]	Cyrany/2014	1/F	26 hours	1 A23	Snare	Deep ulcer
6	[2]	Hammad/2015	31/M	14 hours	5 AAA and 2 AA	Net	Multiple deep ulcer
7	[18]	Kayipmaz/2016	83/F	25 minutes	3 AAA	Snare	Edematous and erythematous mucosa
8	[4]	Tien/2017	17/F	14 hours	1 AAA and 2 AA	Net	Deep ulcer
9	[19]	Yamashiro/2018	61/M	8 hours	13 AA	Net	NA
10	Our case	Our case/2021	45/M	28 hours	8 AA	Snare	Corrosive mucosa
				NA: not available			

In all previous reports, the batteries were removed within three days of ingestion. A Roth net, polypectomy snare, and Dormia basket were used as removal devices. Among them, the basket and net were commonly used to extract small-sized batteries, including AAA batteries. On the other hand, among five cases with ingested AA batteries, similar to our case, a Roth net was used in three cases and a snare was used in two cases. Magnet catheters were used concomitantly in one of the four cases in which the battery was extracted with a snare. In the present case, a Roth net was first selected to retrieve the battery, but it was difficult to store the battery due to its large size. Hence, a polypectomy snare was used, which allowed easy removal of the batteries. Our experience suggests that endoscopic removal should be considered as early as possible if a cylindrical battery is retained in the stomach. In such cases, a polypectomy snare is especially useful for removing relatively large cylindrical batteries, because it can strongly grasp foreign objects with round and slippery surfaces.

## Conclusions

In conclusion, early endoscopic extraction of ingested cylindrical batteries should be considered if they remain in the stomach, due to the increased risk of ulceration and perforation. Contrary to current guidelines, a more aggressive approach should be adopted to remove the batteries, given their potential to cause mucosal damage. On the other hand, when a cylindrical battery has passed the pyloric ring, conservative management with close monitoring is acceptable if there are no clinical symptoms. Additionally, polypectomy snares are useful for the extraction of cylindrical batteries.
